# Travel in Phthisis

**Published:** 1878-09

**Authors:** 


TRAVEL IN PHTHISIS.—James Edward Pollock, M.D., F.R.C.P., in *Medical Times and Gazette:*

1. Never permit any patient to travel who is not in the quiescent stage of disease, or who, in other words, is feverish, with high evening temperature, and the physical signs and conditions already described to you, indicating the continuous form of phthisis. Observe this rule, and you will be successful; break it, and your patient and his friends will not thank you.

2. None of the secondary complications should be present, as continuous or frequent diarrhœa, serious gastric disorder, or laryngeal irritation.

3. Chronic single cavity, with retraction of walls accomplished or proceeding, is favorable for removal to a dry, bracing locality, if the hæmoptysical element be wanting in the case.

4. That form of disease which I have described as diffused deposit in one lung, without much dullness or signs of massing of disease, with pretty large chest, and with moderate emaciation, generally does well on a sea-voyage.

5. A first-stage case already chronic does best for traveling about, with frequent change of residence. The complication with bronchitis or asthma is generally much benefited by change.

6. Persons ought not to travel *at all* with feverish symptoms, with secondary complications, with a large amount of local disease in any stage, with both lungs diseased, with poor digestion and greatly lowered nutrition, or in such a state of weakness or emaciation as to require comforts, peculiar beds or chairs, or varieties of invalid cooking.

